# Fluoride Intensifies Hypercaloric Diet-Induced ER Oxidative Stress and Alters Lipid Metabolism

**DOI:** 10.1371/journal.pone.0158121

**Published:** 2016-06-23

**Authors:** Heloisa Aparecida Barbosa Silva Pereira, Aline Salgado Dionizio, Mileni Silva Fernandes, Tamara Teodoro Araujo, Tânia Mary Cestari, Camila Peres Buzalaf, Flávia Godoy Iano, Marília Afonso Rabelo Buzalaf

**Affiliations:** 1 Department of Genetics and Evolution, Center of Biological Sciences and the Health, Federal University of São Carlos, Washington Luis, km 235,13560–970, São Carlos, São Paulo, Brazil; 2 Department of Biological Sciences, Bauru Dental School, University of São Paulo, Al. Octávio Pinheiro Brisolla, 9–75, 17012–901, Bauru, São Paulo, Brazil; 3 Centro de Ciências da Saúde, Universidade do Sagrado Coração, Rua Irmã Arminda 10–50, 17011–160, Bauru, São Paulo, Brazil; Max-Delbrück Center for Molecular Medicine (MDC), GERMANY

## Abstract

The role of fluoride (F) in oxidative stress is well reported, but its effects on the lipid metabolism has not been completely explored

**Background:**

Here, we evaluated the relationship of diet and F-induced oxidative stress to lipid metabolism in the liver of rats eating normocaloric or hypercaloric diets for two time periods (20 or 60 days).

**Methods:**

Seventy-two 21-day-old Wistar rats were divided into 2 groups (n = 36) based on the type of diet they were eating; each of these groups was then further divided into another two groups (n = 18) based on the time periods of either 20 or 60 days, for a total of four groups. Each of these was divided into 3 subgroups (n = 6 animals/subgroup), dependent on the dose of F administered in the drinking water (0 mg/L(control), 15 mg/L or 50 mg/L). After the experimental period, blood samples and the liver were collected. Plasma samples were analyzed for HDL, cholesterol and triglycerides. Western blots were performed to probe for GRP78, Erp29, SOD2, Apo-E and SREBP in hepatic tissues.

**Results:**

As expected,the expression of target proteins involved in oxidative stress increased in the F-treated groups, especially in liver tissue obtained from animals eating a hypercaloric diet. Most changes in the lipid levels and pathological conditions were seen earlier in the time period, at day 20. The morphometric analyses showed a reduction in steatosis in groups on ahypercaloric diet and treated with 50 mg F/L compared to the control, while no changes were obtained in normocaloric-fed rats. Accordingly, plasma TG was reduced in the F-treated group. The reduced expression of Apo-E in a time- and diet-dependent pattern may account for the particular decrease in steatosis in hypercaloric-fed F-treated rats.

**Conclusions:**

These results suggest that F changes liver lipid homeostasis, possibly because of the induction of oxidative stress, which seems to be higher in animals fed hypercaloric diets.

## Introduction

The widespread use of fluoride (F) has substantially contributed to the reduction in caries prevalence and incidence worldwide [1]. However, the therapeutic window is narrow. While small doses of F may not be enough to allow the maximum preventive effect to be achieved, exposure to higher than optimal F levels has been associated with dental fluorosis [2]. There is substantial body of evidence from *in vivo* and *in vitro* studies showing that very high concentrations of F interact with cellular systems to cause oxidative stress and lipid peroxidation [3]. The extent of its toxicity effect seems to be dependent on the duration of F administration and the age of the animals because of their adaptation to F at younger and older ages [4].

The liver is the main organ responsible for detoxifying organisms [5]. As a consequence, liver disorders lead to problems in all biological systems. Of special interest is alterations in lipid homeostasis because of the imbalance between exogenous-lipid absorption and endogenous lipid biosynthesis, resulting in the generation of lipid droplets [6, 7]. The accumulation of lipid droplets in the form of triglycerides within the liver is known as steatosis, where lipid droplets are found up to 5% of hepatocytes [8]. In fact, hyperglycemia induces both oxidative stress [5, 9, 10, 11] and lipogenesis, thereby increasing the fatty acid contents in hepatic tissue [12].

Work from our laboratory has shown that exposure to high doses of F interferes with lipid metabolism in the liver of rats consuming an AIN-93 diet [13]. The AIN-93 diet is widely used as control diet in studies with rodents, but it is in fact hypercaloric due to its high carbohydrate content. As a consequence, its consumption leads to metabolic disorders, including the accumulation of hepatic fat [14]. Accordingly, the hepatocytes of rats fed this diet presented lipid droplets, but the occurrence was slightly reduced when the animals were chronically exposed to F in the drinking water [13]. At first glance, the ability of F to reduce the deleterious effects of increased energy intake seems contradictory, because F toxicity is reported to provoke lipid peroxidation and oxidative stress [15, 16, 17] that would, in turn, increase steatosis [18, 19].

Previously, F was reported to increase the GRP78 protein, a chaperone that regulates the homeostasis of endoplasmic reticulum (ER) and inhibits apolipoprotein-E (Apo-E) in the liver [13], a protein responsible for the trafficking and delivery of lipids to the organism [20]. They are thought to impair ER-oxidative stress and to reduce fat in the liver, respectively [21]. However, the impact of F-induced ER oxidative stress on the lipid metabolism is unclear.

Knowing that the content of the diet alters the development of steatosis, the aim of this study was to evaluate alterations in lipid metabolism induced by early and late exposure to F in rats eating either a normocaloric or hypercaloric diet and their relation to the oxidative response.

## Materials and Methods

### Animals and treatment

All experimental protocols were approved by the Ethics Committee for Animal Experiments of Bauru Dental School, University of São Paulo (protocol: 037/2011).

Weanling (3-week-old) male *Wistar* rats were randomly distributed into two groups (n = 36/group) receiving hypercaloric (AIN-93M) or normocaloric diets (Presence^®^). Each group was divided into 3 subgroups (n = 6 /subgroup), based on the F (as NaF) concentrations in the drinking water, which were 0 mg/L (control; deionized water), 15 mg/L or 50 mg/L, administered for 20 or 60 days. Both the diet and F were administered concomitantly. The administration of water containing 15 and 50 mg/L F to rats leads to plasma F levels corresponding to those found in humans consuming water containing approximately 3 and 10 mg/L F, respectively [22]. All animals were housed in standard cages (3 animals *per* cage) with chow and water *ad libitum*. Fluoride concentration in both diets was very low (< 1 mg/Kg). Regardless the non-standardization of the F ingestion per animal, only a slight variation in the plasma F was observed among the rats in a previous study [13]. The temperature and humidity in the climate-controlled room, which had a 12 h light/dark cycle, were 23±1°C and 40%–80%, respectively. At the end of study, the animals received an intramuscular injection of anesthetic and muscle relaxant (ketamine chlorhydrate and xylazine chlorhydrate, respectively). Blood samples and livers were collected. Blood was collected by heart puncture, using a heparinized syringe. The levels of triglycerides, HDL (High-Density Lipoprotein), cholesterol and F were analyzed in the plasma. The right lobe of the liver was used for histopathological analysis, while the remaining tissue was assessed for triglyceride quantitation and western blotting.

### Histological assessment

#### Histotechnical processing

The livers were weighed. The right liver lobules (n = 6) were fixed in 10% formalin in phosphate buffer for a week and processed for histology (paraffin embedded). Semi serial sections were cut at 5 μm using a microtome (Microm, model HM 340 E, Germany). Sections were included in slides and stained with hematoxylin and eosin (HE) stain using routine histological protocols.

#### Histological and morphometric analysis

An Axioskop 2 binocular microscope (Carl Zeiss Microscopy GmbH, Jena, Germany) was used to examine and evaluate the histological sections, along with a photographic camera AxioCam HRC (Carl Zeiss Microscopy GmbH, Jena, Germany) and the AxioVision software (Carl Zeiss Microscopy GmbH, Jena, Germany).

#### Morphological analysis—assessment of steatosis

To assess the level of steatosis, a graded score was used. Three blind examiners performed the evaluations. The score was based on the number and size of lipid droplets found in the histological sections of the livers. The qualitative classification of liver lipid content was based on the amount (0 to 5) and size (macro and microdroplets) as follows: none (0), a few microdroplets (1), a moderate amount of microdroplets and few macrodroplets (2), many microdroplets and a moderate amount of macrodroplets (3), microdroplets and agglomerate macrodroplets (4) and many macrodroplets throughout the tissue (5), as displayed in S1 Fig.

The examiners were initially calibrated by evaluating 33% of the samples, which was repeated after 15 days for the purpose of intra-examiner calibration. The histological agreement between groups of paired samples was assessed by using the kappa (*k*) coefficient. Statistical methods were used to assess the agreement between intra- and inter-examiner observations. A kappa coefficient <0.1 was considered as no agreement, 0.11 to 0.4 as fair, 0.41 to 0.6 as moderate, 0.61 to 0.8 as substantial, and 0.81 to 1 as perfect agreement. The *K* coefficient obtained was > 0.8.

#### Morphometric analysis

The morphometrical analysis was conducted blindly using a Zeiss microscope at 40X magnification with reticular integration coupled to an 8X magnification ocular. Ninety fields were selected by systematic sampling [23]. Nonalcoholic steatosis was classified according to the percentage of lipid droplets as follows: normal (≤ 12.9%), mild (13.0–37.9%), moderate (38.0–61.3%) or severe (≥61.4%). The percentage was based on the comparison of morphological data, and this procedure was adapted from Brunt et al. [8].

#### Lipid profile analysis

The level of triglycerides in the plasma and liver was quantified using a commercial kit (Doles, Belo Horizonte, MG, Brazil). For this, 200 mg of liver tissue from each animal was homogenized in 0.5 mL of 0.1% Triton X-100, sonicated for 45 seconds and centrifuged at 4000 rpm for 10 min. The supernatant was obtained for analysis. The HDL and total cholesterol in the plasma were quantified a by commercial kit (Gold Analisa, Presidente Prudente, SP, Brazil). The levels of LDL and VLDL were quantified using the Friedewald formula [24].

### Western blot analysis

The Western blot was performed as previously described [25]. Liver protein extracts were obtained by lyses homogenized tissue in Ripa buffer (0.5% sodium deoxycholate, 0.1% SDS, 1% NP-40) supplemented with protease inhibitors (Roche Diagnostics, Mannheim, Germany). Protein samples (40 μg) were resolved on 10% or 12% Tris-HCl polyacrylamide gels and subsequently transferred to a nitrocellulose membrane. Membranes were probed with commercially available rabbit polyclonal anti-ApoE (1:500 dilution), anti-GRP-78 (1:500) (Abcam, Cambridge, MA, USA), anti-β-tubulin (1:200) (Cell Signaling, Danvers, MA, USA), anti- ERP29 and anti-α-tubulin (1:2000), anti-SREBP -1c and anti-SOD2 (1:500) antibodies (Abcam, Cambridge, MA, USA), followed by HRP-conjugated anti-rabbit antibody (1:10000) and ECL Plus detection reagents (GE Biosciences, Piscataway, NJ, USA). Relative ApoE, GRP-78, β-tubulin, α-tubulin, ERP29, SREBP and SOD2 band densities were determined by densitometrical analysis using the Image Studio Lite software from LI-COR Corporate Offices-US (Lincoln, Nebraska USA). In all instances, density values of bands were corrected by subtraction of the background values. The results were expressed as the ratio of ApoE, GRP-78, ERP29 and SOD2 to that of β-tubulin and SREBP to that of α-tubulin our β-tubulin.

### Statistical analysis

After checking normality and homogeneity, data were evaluated by 2-way ANOVA (experimental time and F concentration as criteria), followed by Tukey´s *post hoc* test using the software Statistica (version 10.0 for Windows, StatSoft. Inc. Tulsa. USA, 2011). The level of significance was set at 5%.

## Results

The weight of the animals increased along time but did not vary as a function of the treatment with F or type of diet (data not shown). Plasma and liver F concentrations increased as a function of F concentration in the drinking water. The animals treated with 50 mgF/L for 60n days receiving normocaloric data had plasma and liver F levels significantly lower when compared with all the other animals treated with 50 mgF/L in the drinking water (S2 Fig)

### Morphological and morphometric analyzes

**Fig 1:**
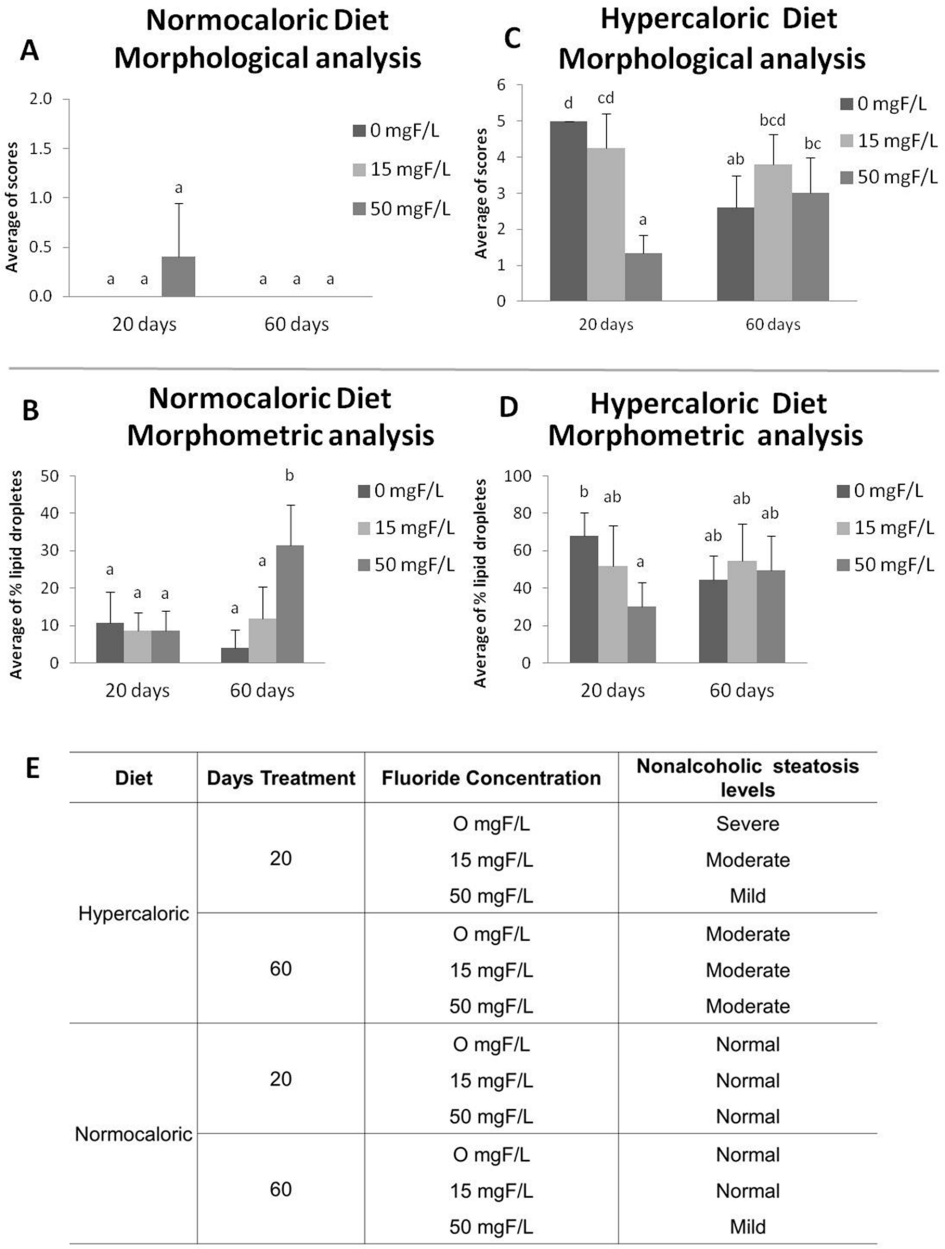
**Histological analysis: A) and C).** Average of scores found in the liver of animals in relation to the type of diet, based on Score photomicrograph of liver. **B) and D)**. Morphometric analysis average of % lipid droplets found in the liver of mice in relation to the diet. **E)** Nonalcoholic steatosis level in each group of treatment: normal ≤12.9%, mild 13–37.9%, moderate 38–61.3% and severe ≥61.4%, based on morphometric analysis.

The hypercaloric diet significantly increased steatosis at 20 and 60 days compared to the normocaloric diet (Fig 1A and 1C). In addition, steatosis was significantly reduced in the hypercaloric diet group treated with 50 mg F/L for 20 days (Fig 1C and 1D). In contrast, the 60-day treatments did not impact the level of steatosis in the hypercaloric groups, regardless of the F concentration (Fig 1C and 1D). In agreement, the morphometric analysis revealed that the nonalcoholic steatosis induced by hypercaloric diet at day 20 was severe, moderate and mild, according to the increasing doses of F: 0, 15 and 50 mgF/L, respectively (Fig 1E). Moreover, the steatosis evaluated on day 60 was moderate, regardless of the F exposure (Fig 1E). However, rats receiving a normocaloric diet and F exposure had normal levels of steatosis, except for the group treated with 50 mg F/L for 60 days, which presented an increase in steatosis (p< 0.05) (Fig 1B and 1E). This was accompanied by mild steatosis in F-treated rats compared to the untreated and normocaloric-fed rats (Fig 1E). In this group, treatment with 50 mg F/L for 20 days caused a significant reduction in steatosis (Fig 1B and 1D). The presence of lipid inclusions in the histopathological examination was confirmed by an osmium tetroxide-based method (S3 Fig)

### Lipid profile: HDL, LDL, VLDL, and total cholesterol

**Fig 2:**
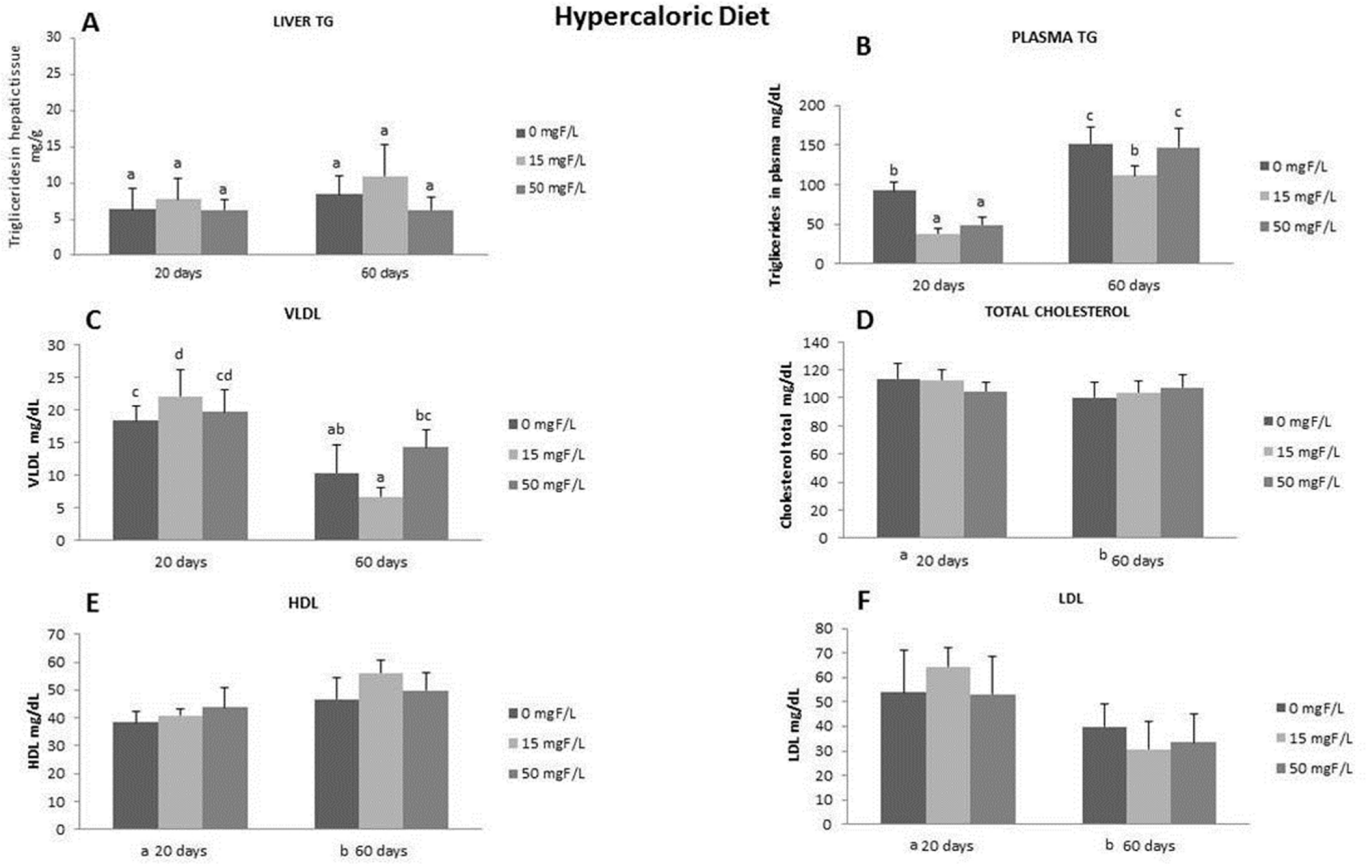
**Lipid profile in animals fed hypercaloric diet**: Means of the lipid profiles in plasma and liver of rats treated with hypercaloric diet receiving different fluoride concentrations in the drinking water for 20 or 60 days. **A)** TG plasma; **B)** TG liver, **C)** VLDL, **D)** Cholesterol, **E)** HDL, **F)** LDL. For each variable, distinct upper case superscripts indicate significant differences among the groups (2-way ANOVA and Tukey’s test, p<0.05).

**Fig 3:**
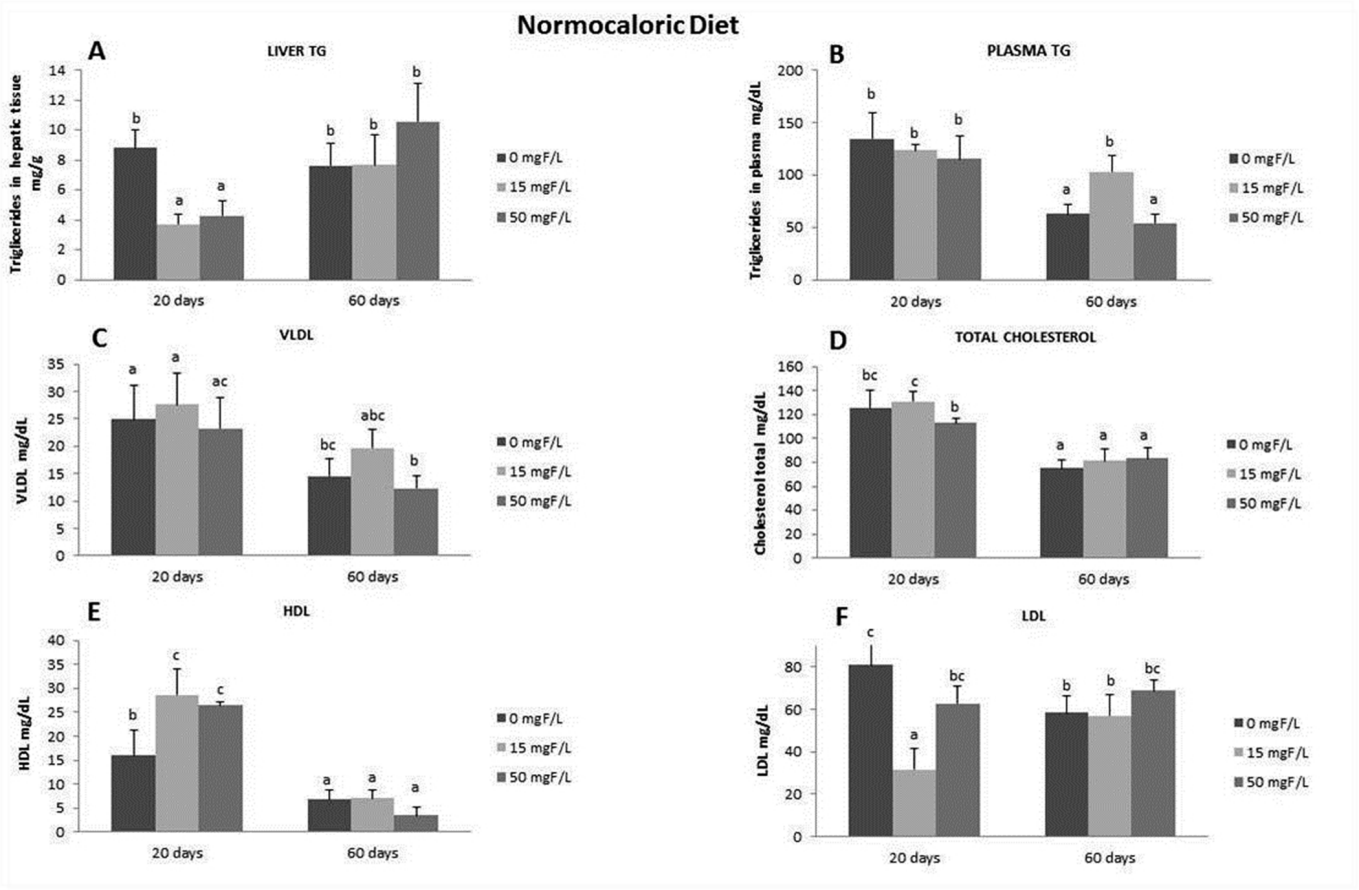
**Lipid profile in animals fed normocaloric diet**: Means of the lipid profiles in plasma and liver of rats treated with normocaloric diet receiving different fluoride concentrations in the drinking water for 20 or 60 days. **A)** TG plasma; **B)** TG liver, **C)** VLDL, **D)** Cholesterol, **E)** HDL, **F)** LDL. For each variable, distinct upper case superscripts indicate significant differences among the groups (2-way ANOVA and Tukey’s test, p<0.05).

To verify the role of F in promoting lipid metabolism changes, which may depend on the diet type, the F dose, and the duration of exposure, we assessed the lipid parameters and triglycerides (TG) in the plasma and TG in the liver (Figs 2 and 3). While the level of hepatic TG of the animals receiving hypercaloric diet for 20 and 60 days did not change among the groups (Fig 2A), the plasma TG level significantly increased within the period (Fig 2B). The reduced extension of steatosis promoted by 20 days of 50 mg F/L treatment in the hypercaloric-group was accompanied by decreases in the level of plasma TG, whereas doses of 15 mg F/L significantly reduced the level of TG at day 60 (Fig 2B). The level of VLDL was increased in the hypercaloric-fed rats treated with 15 mg F/L compared to the control group at 20 days, which was significantly higher when compared to the same group (Fig 2C). The levels of total cholesterol, HDL and LDL were not altered in the hypercaloric-fed groups, regardless of the presence and time of F exposure (Fig 2D, 2E and 2F, respectively). Regarding the lipid profile in normocaloric rats, treatment with F decreased the level of liver TG at day 20, while it did not change the plasma TG, which, in turn, was enhanced at day 60 (Fig 3A and 3B). In addition, while the levels of VLDL and total cholesterol were unaltered (Fig 3C and 3D), the level of HDL was increased in F-treated normocaloric groups at day 20 (Fig 3E). Instead, the levels of LDL were lower in the 15 mg F/L-group at this time point (Fig 3F).

### Western Blot

**Fig 4:**
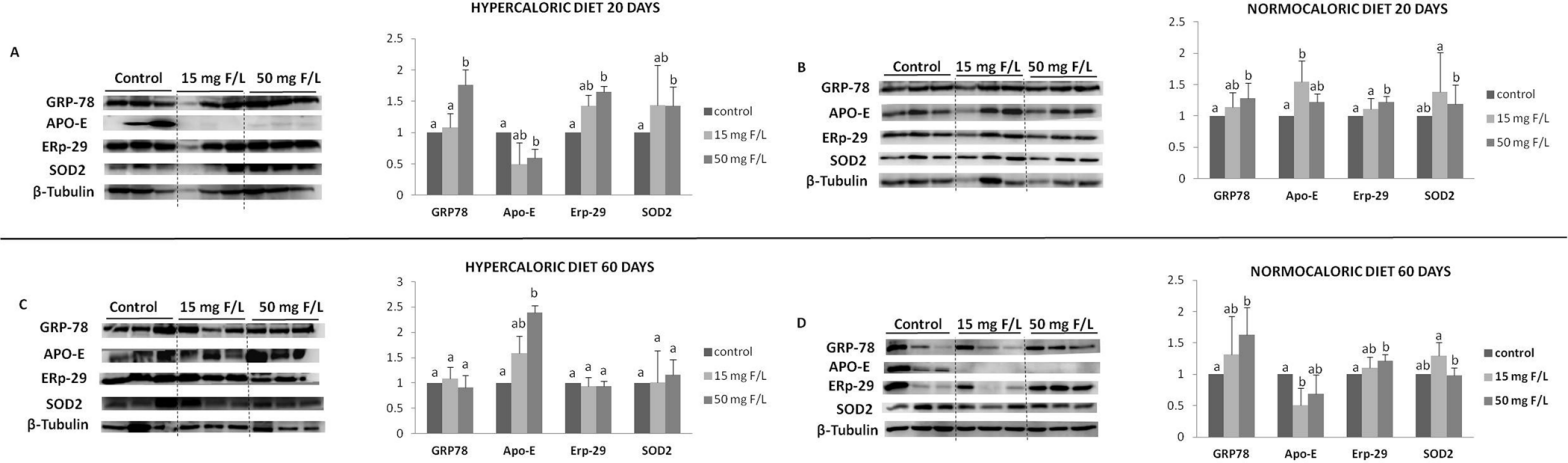
Representative expression of proteins GRP-78, APO-E, SOD2, ERP-29 and of the constitutive protein β-tubulin in samples of individual animals (n = 3) from each group. Densitometric analysis was performed for 6 animals per group. **A.** Protein expression in the liver of rats in the group treated with the hypercaloric diet for 20 days; **B.** Protein expression in the liver of rats treated with the normocaloric diet for 20 days; **C.** Protein expression in the liver of rats in in the group treated with the hypercaloric diet for 60 days; **D.** Protein expression in the liver of rats treated with the normocaloric diet for 60 days. Densitometry was analyzed using the software *Image Studio Lite*. Vertical bars represent standard deviations. For each condition, distinct superscripts denote significant differences among the groups (ANOVA and Tukey’s test, p<0.05, n = 6).

**Fig 5:**
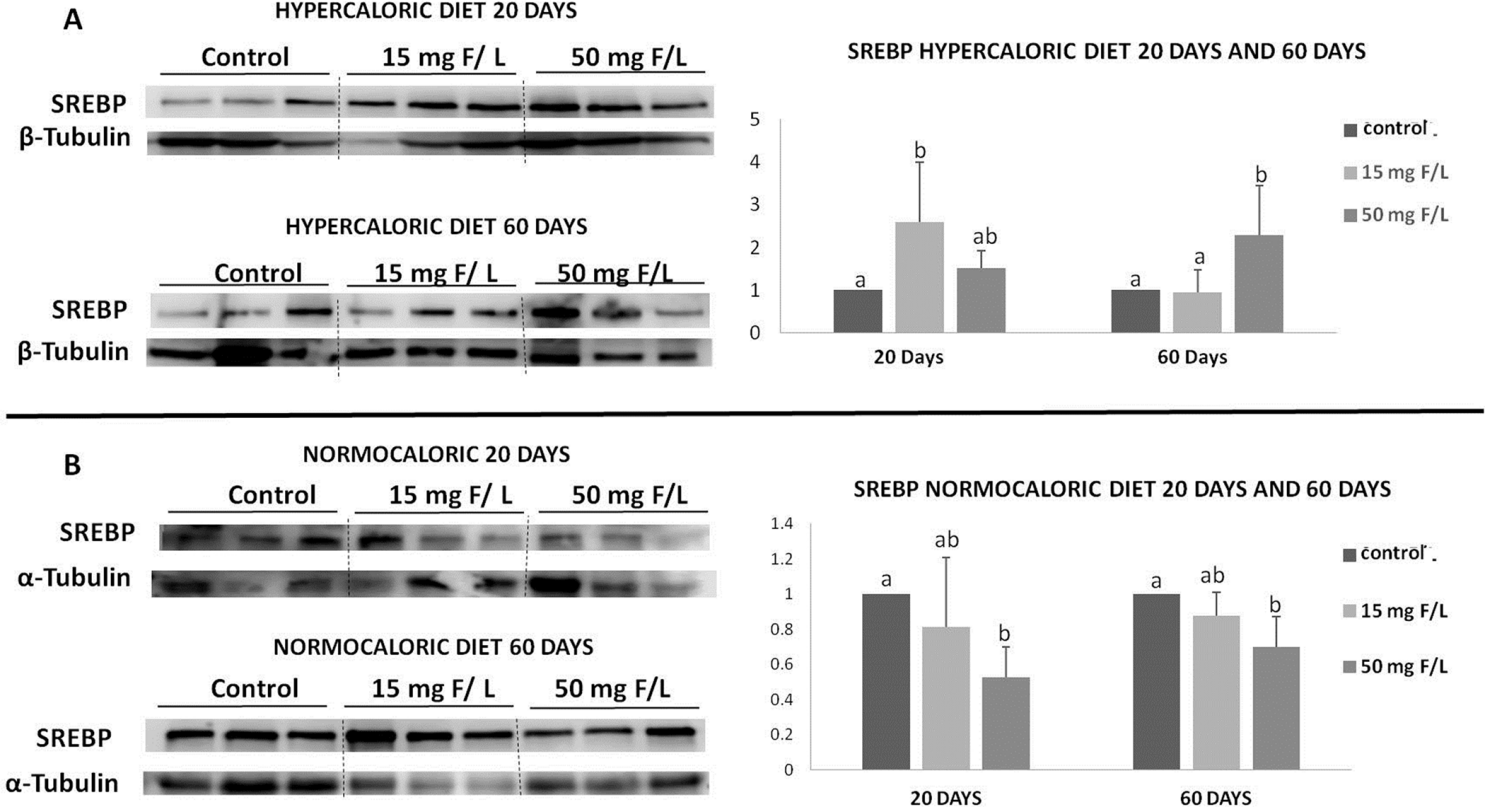
Representative expression of proteins SREBP, and of the constitutive protein β-tubulin ou α-tubulin in samples of individual animals (n = 3) from each group. Densitometric analysis was performed for 6 animals per group. **A.** Protein expression in the liver of rats in the group treated with the hypercaloric diet for 20 and 60 days; **B.** Protein expression in the liver of rats treated with the normocaloric diet for 20 and 60 days. Densitometry was analyzed using the software *Image Studio Lite*. Vertical bars represent standard deviations. Distinct superscript denote significant differences among the groups (ANOVA and Tukey’s test, p<0.05, n = 6).

To investigate the mechanism by which F alters the lipid profile, key proteins related to lipid metabolism and oxidative stress were identified and quantified by western blot analysis. GRP78 is related to *de novo* lipogenesis, Apo-E is involved in lipid transport and delivery to the liver [20] and SREBP participates in the activation of TG synthesis [21]. Increases in the expression level of SOD are related to oxidative stress [26], while ERp29 indicates endoplasmic reticulum-specific oxidative stress [27]. The hypercaloric diet significantly increased the expression of GRP78 and ERp29 expression in the 50 mg F/L-treated groups compared to the untreated group at day 20 (Fig 4A). Moreover, SOD was upregulated in F-treated rats, regardless of the dose. Apo-E expression was down- and upregulated in hypercaloric animals that received F on days 20 and 60, respectively (Fig 4A and 4B). However, in animals fed a normocaloric diet, exposure to F significantly increased GRP78, regardless of the time point, while Apo-E expression was up- and down-regulated after 15 mg F/L treatment at days 20 and 60, respectively (Fig 4C and 4D). The levels of ERp29 were enhanced in rats receiving 50 mg F/L at both 20 and 60 days (Fig 4C and 4D). In addition, SOD expression was increased in the 15 mg F/L-treated group at day 60 (Fig 4D). The hypercaloric diet induced an increase in the level of SREBP in the 15 mg F/L-treated group at day 20 and in the 50 mg F/L-treated group at day 60 day (Fig 5D). However, for the normocaloric diet groups, the 50 mg F/L-treated group had a decreased level of SREBP at both periods of time (Fig 5E).

## Discussion

The animal model chosen in the present study has been widely used in studies involving F metabolism [13, 17, 28, 29]. The F concentrations added in the drinking water were selected to simulate doses considered toxic to humans, which are above 2 mg/L (10 mg/L for rats) [30].

Recently, several studies revealed the effects of F on lipid metabolism [31–35].To our knowledge, this is the first study to highlight the mechanisms by which F alters hepatic lipid metabolism depending on the time of exposure and the content of the diet. In addition, this response may be secondary to the well-known F-induced ER oxidative stress [3, 15, 17, 34–37]. Conclusions based on our results can be inferred after observing a reduction in liver lipid droplets after treatment with F for 20 days in hypercaloric-fed rats. This effect was not seen in normocaloric-fed animals. In fact, the lipid droplets visualized had different diameters, a characteristic also reported in other studies that is partly attributed to the fact that these fat droplets increase in size over time [38]. We standardized a score for measuring lipid droplets, and animals that received the hypercaloric diet had an average score of 4 in terms of their lipid droplets and had severe steatosis, while animals that received the normocaloric diet had an average score of 1 in terms of their lipid droplets and had normal steatosis. Similar data were found in another study in which animals were fed high-fat and normal diets [39]. In addition, the consumption of a hypercaloric diet significantly increased steatosis, which declined upon the time of exposure. The reduction in the percentage of lipid droplets in the liver regardless of the diet content at day 60 might be due to the metabolic adaptation of animals to the diet [40]. This results in homogeneity among the groups, as reported in previous studies [4, 13]. However, the levels of plasma cholesterol were increased in rats fed the hypercaloric diet in the long term when compared to the normocaloric diet-fed rats. Surprisingly, rats consuming the normocaloric diet presented increased nonalcoholic steatosis at day 60 after treatment with F at 50 mg/L, revealing its dose- and time- dependent manner. Several works have shown that F can have a dual effect, either protective or toxic, depending on the dose and time of exposure [3, 4, 17, 22, 28]. In relation to lipid metabolism in the liver, we have previously shown that exposing rats fed with an AIN-93M diet to F for 60 days induced a slight decrease in liver lipid droplets, a response partially attributed to statistically significant reduction in the Apo-E expression [13]. The dependency on the time of exposure to F becomes clear when the results regarding plasma TG are considered. For the animals fed the hypercaloric diet, the administration of 15 mg F/L significantly reduced plasma TG levels compared to the control group, regardless of the exposure time. However, for the animals treated with 50 mg F/L only for the short time, a significant reduction in plasma TG was observed compared with the control, which did not occur in the long-term exposure group. This might be due to the adaptive mechanisms of the organism to F [4] that are triggered by high doses of this ion but not by lower ones. In the present study, F did not affect total cholesterol levels, regardless of the time of exposure or the type of diet administered. Some reports demonstrated an increase in cholesterol levels with very high doses of F, but these data are irrelevant because this is hardly found in practice [32] or the animals were older when treatment with F started, revealing an adaptive response with increased exposure time [41]. When the cholesterol fractions were evaluated, it was observed that administration of a lower dose of F in the short term with a normocaloric diet showed a beneficial effect by significantly increasing the level of HDL and decreasing the level of LDL compared with the control. This had an impact on the concentration of TG in the liver, which also significantly decreased in this group. It should be highlighted that the highest dose of F did not alter either the level of HDL or LDL.

F and the type of food are considered two stress factors that trigger oxidative responses. Alterations in lipid percentage contents lead to oxidative stress as well as to changes in calcium homeostasis [42, 43]. In addition, the oxidative stress in response to F is commonly reported in the literature [17, 44]. We hypothesized that oxidative stress could be a key factor in triggering changes in the lipid metabolism. To address this, the expression of proteins related to oxidative stress and lipid and calcium trafficking were analyzed. Our data provide mechanistic insights on F-induced alterations in the lipid profile. The increased expression of GRP78 and ERP29, both proteins related to oxidative stress [21, 27, 45], were found when the highest dose of F was administered to hypercaloric diet-fed rats at day 20. In addition, the expression level of the antioxidant protein SOD2 also increased, probably in attempt to counteract the stress generated by F [20]. However, normocaloric rats treated with F presented an increased expression of GRP78, suggesting lower oxidative stress. No comparative analysis of these markers in the different time and type of diet groups a is allowed because the samples were loaded on different membranes. Thus, our results indicate that F induces oxidative stress, which is modified by the type of diet and time of administration. Some studies indicate that ER stress can alter the accumulation of cholesterol in the liver, which in turn inhibits Apo-E [46]. However, we did not see alteration in the levels of plasma cholesterol, which make us wonder whether the reduction of Apo-E is directly induced by F or by the up-regulation of the expression of GRP78, which in turn may reduce ER stress. We showed that Apo-E expression was significantly reduced following treatment with F and consumption of a hypercaloric diet at the earlier time point. In this same condition, GRP78, ERp29 and SOD-2 were upregulated. In contrast, when the treatment with F was associated with the normocaloric diet, no differences were observed for the Apo-E expression, regardless of the higher expression of GRP78. It has been shown that GRP78 inhibits ER stress, reducing hepatic steatosis in mice [21]. Moreover, ER stress-dependent hepatic steatosis was reduced in livers of ApoE-deficient mice, showing that steatosis can be reduced when the availability of lipoproteins to deliver fat to liver is lower [20, 46].

Previous work from our lab showed that a reduction of Apo-E was observed in animals treated for 60 days with 50 mg F/L and fed a noncommercial hypercaloric diet [13]. However, the levels of Apo-E levels were not different at day 60 in this study. This contradictory result made us to believe that the commercial and in-house prepared AIN-93M diets used in the first and second studies, respectively, are critical for lipid content delivery. In agreement, the in-house AIN-93M diet promoted a greater score for the lipid droplets in the liver.

In contrast, the reduction in SREBP expression was found in the normocaloric diet and F-treated group. This is consistent with increased GRP78, which was shown to inhibit *de novo* lipogenesis [21], leading to a reduction in liver TG. Although liver TG was reduced, no differences were found in the steatosis scores. It was demonstrated that GRP78 expression inhibits insulin- and ER stress-induced SREBP-1c activation and reduces hepatic steatosis in mice [21].

**Fig 6:**
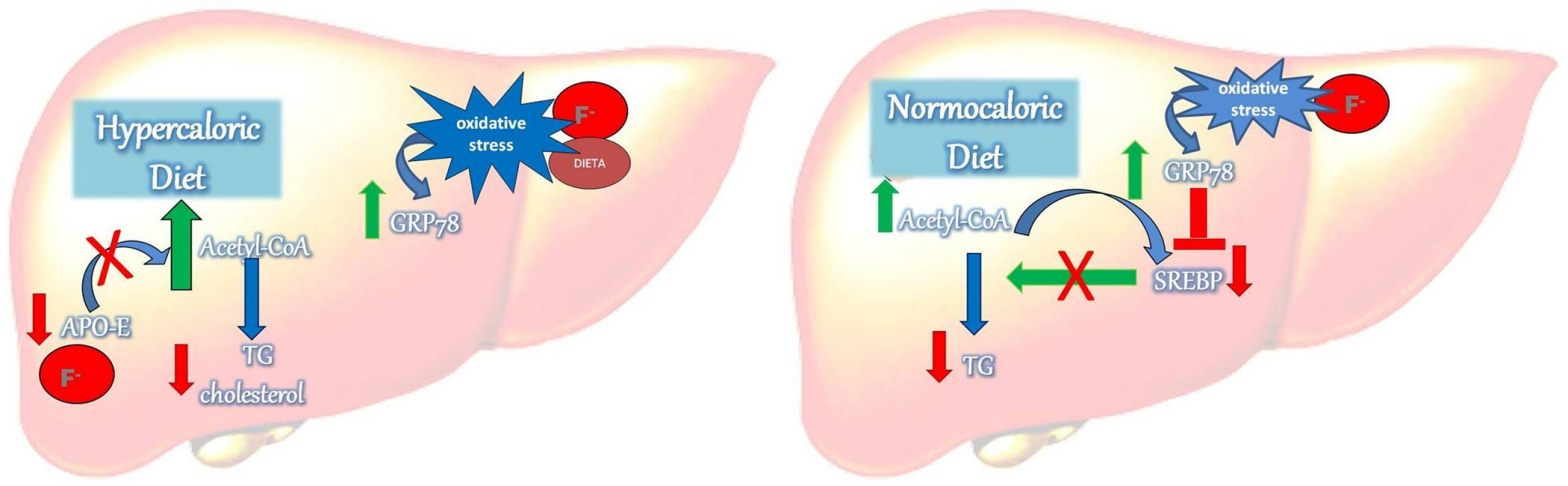
Proposed mechanism by which fluoride affects lipid metabolism depending on the type of diet consumed and time of exposure. The diet-dependency effects of fluoride in lipid metabolism are observed strictly at earlier periods and are related with ER oxidative stress. Upon consumption of normocaloric diet, fluoride-induced oxidative stress leads to increase of GRP78, which in turn reduces SREBP, thus deactivating TG synthesis. However, in the presence of hypercaloric diet, fluoride exposition induces oxidative stress highlighted by GRP78, ERp29 and SOD2 increases, which in turn, by undetermined mechanism, leads to reduction of Apo-E, which diminishes precursors of Acetyl–Coa and leads to less formation of liver TG.

Based on the above-mentioned results regarding the diet-dependent effects of F on lipid metabolism observed strictly at earlier periods and its relationship with ER oxidative stress, we proposed a mechanism to explain these effects (Fig 6). Upon the consumption of a normocaloric diet, F-induced oxidative stress leads to an increase in GRP78, which in turn reduces SREBP, thus deactivating TG synthesis. However, with the consumption of a hypercaloric diet, F exposition induces oxidative stress highlighted by increases in GRP78, ERp29 and SOD2 expression, which in turn, by an undetermined mechanism, leads to a reduction in Apo-E, which diminishes the precursors of Acetyl–CoA and leads to decrease in the formation of liver TG. Here, the dependency of SREPB was not revealed.

In summary, reductions in lipid metabolism induced by F seem to be linked to ER stress, a phenomenon that is more evident in the consumption of a hypercaloric diet. These data provide new insights on the current understanding of the mechanism by which F induces steatosis and dyslipidemia. The importance of this conclusion is highlighted by the fact that the administration of F, at similar doses and times of exposure to the ones employed in the present study, together with a normocaloric diet, increases insulin sensitivity, which may have a beneficial effect in rats affected by diabetes, a disease related to lipid and carbohydrate metabolism [28].

## Supporting Information

S1 Fig Photomicrographs showing score of lipid droplets in liver. **(A)** Score 0 shows absence of lipid droplets; **(B)** Score 1 shows few and small lipid droplets (diameter ≤ 3μm, dashed circle) sparse into hepatocytes; **(C)** Score 2 shows few large lipid droplets (diameter > 3 μm, yellow arrow) and high amount of small lipid droplets (dashed circle), **(D)** Score 3 shows high amount of small (dashed circles) and moderate large lipid droplets (yellow arrows), **(E)** Score 4 shows agglomerates of large lipid droplets (yellow arrows) with some measuring around 20 μm in diameter (black arrows) and small lipid droplets (dashed circle), and **(F)** Score 5 exhibits high amount of large lipid droplets (yellow arrow) with some measuring around 20 μm in diameter (black arrows). HE
(TIF)

S2 Fig Concentration of fluoride in **(A)** plasma (mg/L) and **(B)** liver (μg/g) of rats receiving normocaloric or hypercaloric diet and treated with F (15 and 50 mg/L) in the drinking water for 20 and 60 days. Distinct superscripts denotes significant differences among the groups (3-way ANOVA and Tukey’s test, p<0.05). n = 6.
(TIF)

S3 Fig Photomicrography of liver impregnated with osmium tetroxide: Normocaloric diet gruop showed few and small (diameter ≤ 3μm) lipid droplets (dashed circle) into hepatocytes; Group treated with hypercaloric diet and 0 mgF/L exhibited high amounts of small (dashed circle) and large (> 3 μm) lipid droplets with some measuring about 20 μm of diameter (white arrows); Group treated with hypercaloric diet and 15 mgF/L presented high amount of lipid droplets with diameter ≤ 10 μm (arrow heads); Group treated with hypercaloric diet and 50 mgF/L showed small lipid droplets (dashed circle) similar to those observed in group treated with the normocaloric diet, but with a higher number of droplets. (TIF)
