# Supplementary figures and images for: Fluoride Intensifies Hypercaloric Diet-Induced ER Oxidative Stress and Alters Lipid Metabolism
Source: PLoS One. 2016 Jun 23;11(6):e0158121. doi: 10.1371/journal.pone.0158121 (PMC4919043; doi:10.1371/journal.pone.0158121)

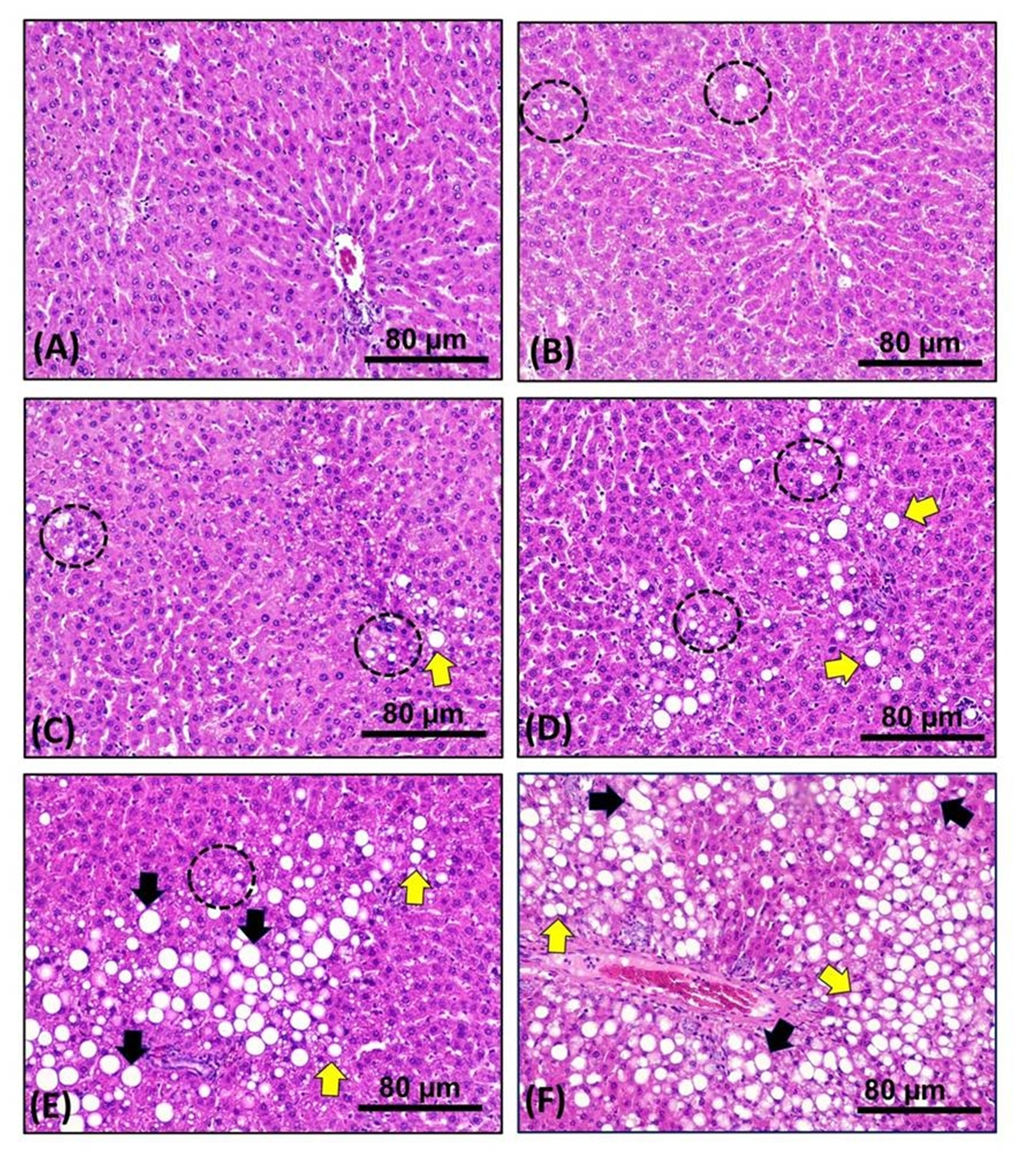

Supplement: S1 Fig — (A) Score 0 shows absence of lipid droplets; (B) Score 1 shows few and small lipid droplets (diameter ≤ 3μm, dashed circle) sparse into hepatocytes; (C) Score 2 shows few large lipid droplets (diameter > 3 μm, yellow arrow) and high amount of small lipid droplets (dashed circle), (D) Score 3 shows high amount of small (dashed circles) and moderate large lipid droplets (yellow arrows), (E) Score 4 shows agglomerates of large lipid droplets (yellow arrows) with some measuring around 20 μm in diameter (black arrows) and small lipid droplets (dashed circle), and (F) Score 5 exhibits high amount of large lipid droplets (yellow arrow) with some measuring around 20 μm in diameter (black arrows). HE (TIF) [file pone.0158121.s001.tif]

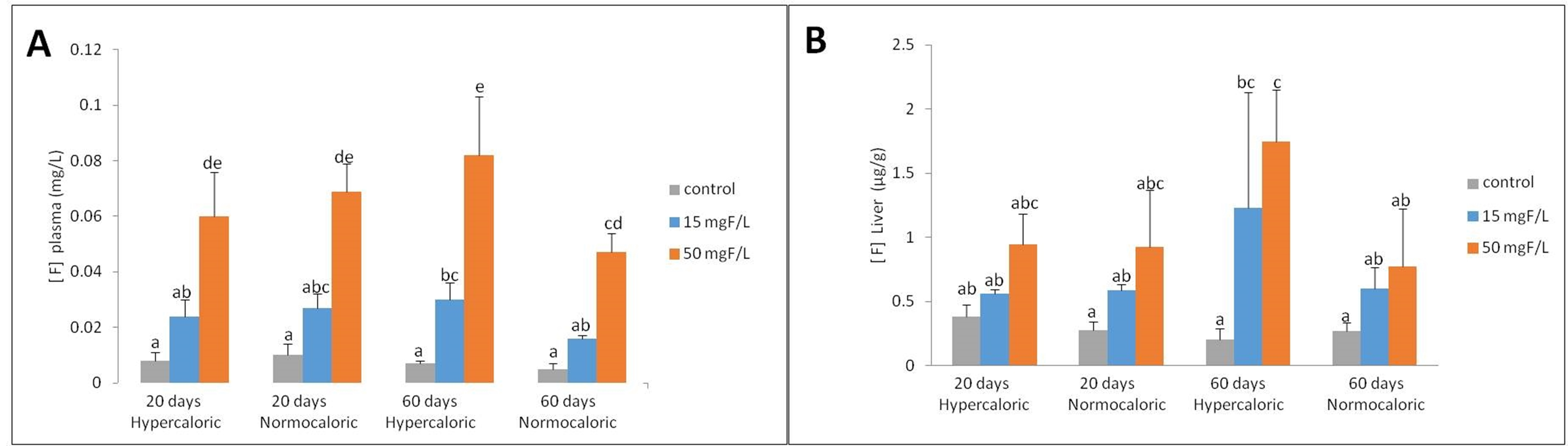

Supplement: S2 Fig — Concentration of fluoride in (A) plasma (mg/L) and (B) liver (μg/g) of rats receiving normocaloric or hypercaloric diet and treated with F (15 and 50 mg/L) in the drinking water for 20 and 60 days. Distinct superscripts denotes significant differences among the groups (3-way ANOVA and Tukey’s test, p<0.05). n = 6. (TIF) [file pone.0158121.s002.tif]

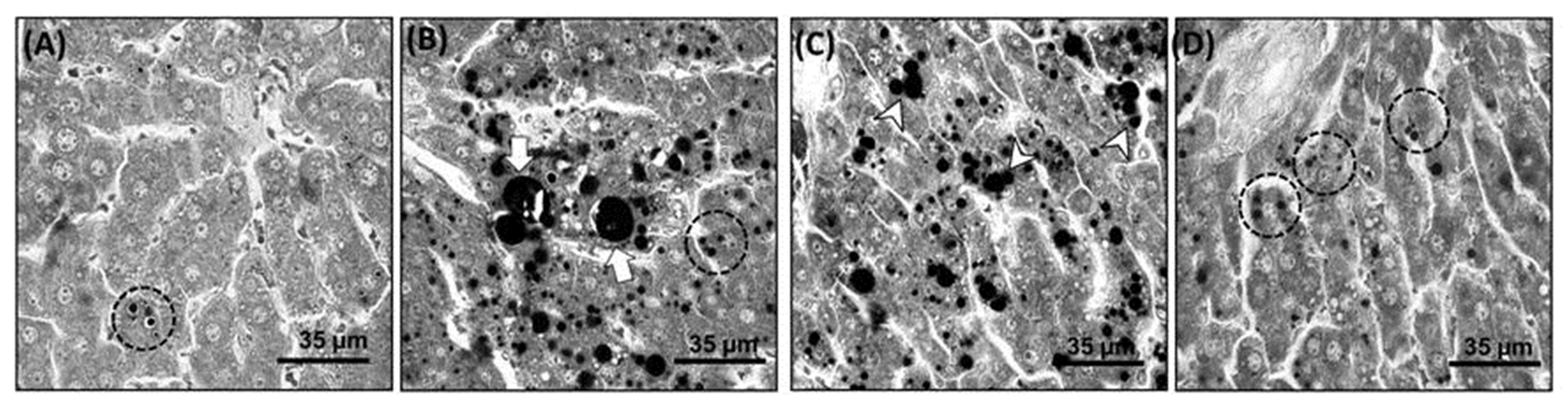

Supplement: S3 Fig — (TIF) [file pone.0158121.s003.tif]
